# Suprachoroidal triamcinolone acetonide for the treatment of macular edema associated with retinal vein occlusion: a pilot study

**DOI:** 10.1186/s12886-023-02808-5

**Published:** 2023-02-10

**Authors:** Boushra M. Ali, Arwa M. Azmeh, Nawras M. Alhalabi

**Affiliations:** Department of Ophthalmology, Faculty of Medicine, Damascus University, Fayez Mansour St, Damascus, Syria

**Keywords:** Suprachoroidal space, Choroid, Macular edema, Retinal vein occlusion, Triamcinolone acetonide

## Abstract

**Background:**

Suprachoroidal Drug Delivery has emerged in recent years as a novel promising approach, which may help address the clinical unmet needs in the management of Retinal Vein Occlusion (RVO) associated Macular Edema (ME). In this study, we aim to evaluate the feasibility in regard of the potential efficacy and safety of suprachoroidal injection of Triamcinolone Acetonide (TA) using a microinjector as a mono-treatment of ME due to RVO.

**Methods:**

This trial included 16 eyes of 16 patients with RVO associated ME presenting to the department of ophthalmology, Al Mouwasat university hospital, Syria. 4 mg of preserved TA was injected suprachoroidally 4 mm away from the inferotemporal limbus using a patient-customized microinjector. After injection, patients were followed after 1 week then monthly for 3 months.

Primary outcome measures included the percentage of participants with best-corrected visual acuity (BCVA) gain≥15 letters and increased intraocular pressure (IOP) ≥ 20 mmHg in months 1,2, and 3, secondary measures included mean change from baseline BCVA, central subfield thickness (CST), and IOP through each of the follow-up points in addition to other measures.

**Results:**

After injection, BCVA gain≥15 letters occurred in 68.7, 62.5, 50, 50% of patients at week 1 and through months 1,2 and 3 respectively, the mean BCVA improved significantly by 16.4, 16, 14.4, and 11.9 letters (*p*-value< 0.0005) at week 1 and months 1,2 and 3 respectively. This visual gain was associated with a significant reduction of CST by 290.94 ± 181.76 (week-1) (*p*-value< 0.0005), 274.31 ± 184.60 (month-1) (*p*-value< 0.0005), 183.50 ± 165.61 (month-2) (*p*-value = 0.006) and 137,75 ± 156.25 μm (month-3) (*p*-value = 0.038). We reported one case of increased IOP ≥ 20 mmHg in the first month that decreased in the second month. The mean change of IOP readings was not statistically significant, with an increase ranging from 0.75 mmHg after the first week (*p*-value = 0.09) and 0.5 mmHg after 3 months (*p*-value = 0.72).

**Conclusion:**

This study suggests that suprachoroidal TA could be well tolerated and efficacious as a mono-treatment of RVO associated ME. Future clinical trials are required to confirm its longer-term safety and efficacy and to compare this efficacy with the other therapeutic options.

**Trial registration:**

This study was retrospectively registered at clinicaltrials.gov (ID: NCT05038072) on 08/09/2021.

This article was published as a preprint on 22/06/2022. 10.21203/rs.3.rs-1701105/v1.

## Introduction

Retinal vein occlusion (RVO) is the second most frequent vision-threatening vascular retinopathies following diabetic retinopathy [1]. It affects 28.06 million people with an estimated prevalence of 0.77% worldwide [2]. Based on the site of occlusion, RVO can be broadly categorized as branch retinal vein occlusion (BRVO), Hemi-retinal vein occlusion (HRVO), and central retinal vein occlusion (CRVO). Macular edema (ME) is a frequent complication of all RVO types and a major cause of RVO-associated visual impairment [1, 3]. Management of RVO associated ME still poses a therapeutic challenge taken into consideration its complicated etiopathogenesis [4–6]. Despite that intravitreal injection of antiangiogenics and steroids have dramatically improved the visual and anatomical outcomes [7–15], optimal management of RVO associated ME is still hindered by the presence of non-responders, tachyphylaxis, rebound phenomenon, high re-injections [16–18], and adverse events rate [13–15], which in turn represent an increasing psychosocial and economic burden and underscore the significance of seeking novel approaches to formulate treatment strategies.

The suprachoroidal space (SCS) is a potential expandable space between the choroid and the sclera that extends over the entire circumference of eye’s posterior segment from the ciliary body rearward [19, 20]. Delivery of therapeutics into the SCS provides a novel alternative approach that has theoretical appeal, as it dominantly targets chorio-retinal tissues with posterior and circumferential distribution while relatively restraining delivery to the unaffected anterior segment and the vitreous chamber, thus reducing hazards associated with off-target effects which enhances its safety profile [20]. This hypothesis was further proven by many preclinical and clinical studies through microinjectors [21–25], which has been shown to provide a safe, minimally invasive, and reliable method of targeting SCS [25]. In addition, extended duration of action and desirable pharmacokinetic properties have been reported for small molecule suspensions including Triamcinolone Acetonide (TA), with the potential to lessen the burden of treatment [21].

The present study evaluated the feasibility in regard of potential efficacy and safety of suprachoroidally injected Triamcinolone Acetonide using a patient-customized microinjector as a monotreatment of Macular Edema (ME) due to Retinal Vein Occlusion (RVO).

This study was retrospectively registered at clinicaltrials.gov (ID: NCT05038072) on 08/09/2021.

## Method

### Study design

This interventional prospective study included 16 eyes of 16 patients with macular edema associated with retinal vein occlusion who attended the outpatient eye clinic of Al Mouwasat University Hospital between July 2019 and November 2020 after being reviewed and approved by the Institutional Review Board (IRB) of the Faculty of Medicine, Damascus University, Syria (approval code 1969/S.M).

All procedures were performed in accordance with the tenets of the Helsinki Declaration. All participants provided written informed consent after discussing the technique of the procedure, its benefits and potential risks, alternative treatment approaches, carried-out examinations, and follow-up schedules. This study was retrospectively registered at clinicaltrials.gov (ID: NCT05038072) on September 8, 2021.

### Patient eligibility and exclusion criteria

This study included male and nonpregnant female patients older than 18 years of age who have a clinical diagnosis of RVO associated with decreased best-corrected visual acuity (BCVA) in Early Treatment Diabetic Retinopathy Study (ETDRS) letter score in the study eye of between 20 (0.05 Snellen equivalent) and 75 (0.63 Snellen equivalent) and increased central subfield thickness (CST) of ≥310 mm measured by spectral-domain optical coherence tomography (SD-OCT). Exclusion criteria included either of the following in the study eye: (1) IVT injection of anti VEGF: Bevacizumab (Avastin; Genentech, South San Francisco, CA, USA/Roche, Basel, Switzerland) or Ranibizumab (Lucentis; Genentech Inc., South San Francisco, CA, USA) within 1 month or Aflibercept (Eylea®; Bayer HealthCare Pharmaceuticals, Berlin, Germany and Regeneron Pharmaceuticals Inc., Tarrytown, NY, USA) within 2 months; (2) Either intraocular or periocular injection of Triamcinolone Acetonide within 3 months, dexamethasone implant (Ozurdex, Allergan, Dublin, Ireland) within 6 months, Retisert (Bausch and Lomb, Bridgewater, NJ) within 1 year, or fluocinolone acetonide implant (Iluvien, Alimera Sciences, Alpharetta, GA) within 3 years; (3) macular laser photocoagulation treatment; (4) Use of nonsteroidal anti-inflammatory eye drops within a month; (5) IOP > 22 mmHg, or history of steroid induced ocular hypertension; uncontrolled glaucoma; (6) Past vitreoretinal or glaucoma surgery; (7) any significant media opacity that could impede assessment of the retina or any other ocular condition that could impair visual acuity other than RVO; or (8) uncontrolled systemic disease that could hinder follow-up, immunodeficiency, or any other systemic contraindication for steroids.

All patients underwent a comprehensive ophthalmic evaluation, including a BCVA test using a Snellen chart whose resultant measures were converted to ETDRS letter score for statistical analysis, IOP measurements using Airpuff TOPCON Computerized Tonometer CT-80, and a thorough anterior and posterior segments examination using slit lamp biomicroscopy and indirect ophthalmoscopy. CST was measured using spectral domain optical coherence tomography (SD-OCT) (Heidelberg Spectralis; Heidelberg Engineering, Germany). Anterior segment optical coherence tomography (AS-OCT) was also performed to measure the thickness of the sclera and surrounding tissues at the injection point (4 mm away from the inferotemporal limbus).

### Surgical procedure

#### Microinjector preparation

The used microinjector, which was prepared for each patient individually, consisted of 1 cc 27-gauge insulin syringe (1 ml Syringe with a hypodermic needle 27G X 1/2″; Secured Medical Direction UK Co., Ltd., London, UK) and a silicone sleeve that was cut from a 26-gauge intravenous catheter. This sleeve was inserted into the 27G needle so that the protruded free part of the needle represents the effective length, which is consistent with the previously measured thickness of the sclera and surrounding tissues at the injection point. The measurement of the silicone sleeve’s length was performed under the microscope using a vernier caliper.

#### Injection technique

Patients were instructed to apply topical fluoroquinolone eye drops q.i.d for 3 days before injection. The patient’s pupil was dilated pre-injection. We used preservative containing Triamcinolone Acetonide (TA) (1 mL vial of TRIACORT® 40 mg\mL, Pharmatex Italia). After sedimentation for 30 minutes, the supernatant was discarded, and TA was then diluted again to 1 mL using BSS. The suprachoroidal injection procedures were conducted in the operating room under an operating microscope applying complete aseptic techniques. Before injection, 0.5% proparacaine hydrochloride eye drop was applied topically followed by application of 10% povidone-iodine to the skin of the periocular area, lids, and eyelashes and 5% povidone-iodine into the conjunctival sac for 3 minutes before injection. 0.1 ml (4 mg) of the prepared TA was injected suprachoroidally 4 mm from the limbus in the inferotemporal quadrant perpendicular to sclera using the microinjector. After the injection, pressure was applied on the injection site using a cotton tip for 1 minute to avoid the leakage of Triamcinolone from the injection point.

#### Postoperative care and follow-up

**Fig. 1:**
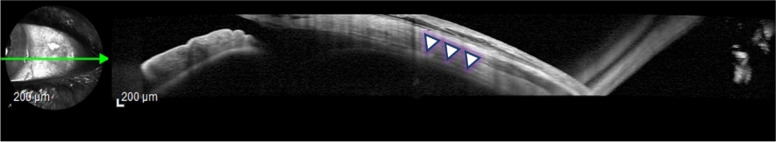
Anterior segment optical coherence tomography (AS-OCT) scan of the temporal anterior sclera immediately after injection showing triamcinolone acetonide localized in the suprachoroidal space (arrowheads)

Topical 5% povidone-iodine was reapplied immediately after injection; intraocular pressure was checked out and indirect fundoscopy was also performed immediately after the injection to detect the occurrence of triamcinolone penetration into the vitreous or any of the immediate side effects and the eye was patched for a few hours. Patients were instructed to apply antibiotic fluoroquinolone drops q.i.d for 3 days and were followed up after a week, then monthly for 3 months. At each visit, a comprehensive ophthalmic evaluation and SD-OCT were carried out Fig. 1.

### Outcome measures

**Fig. 2:**
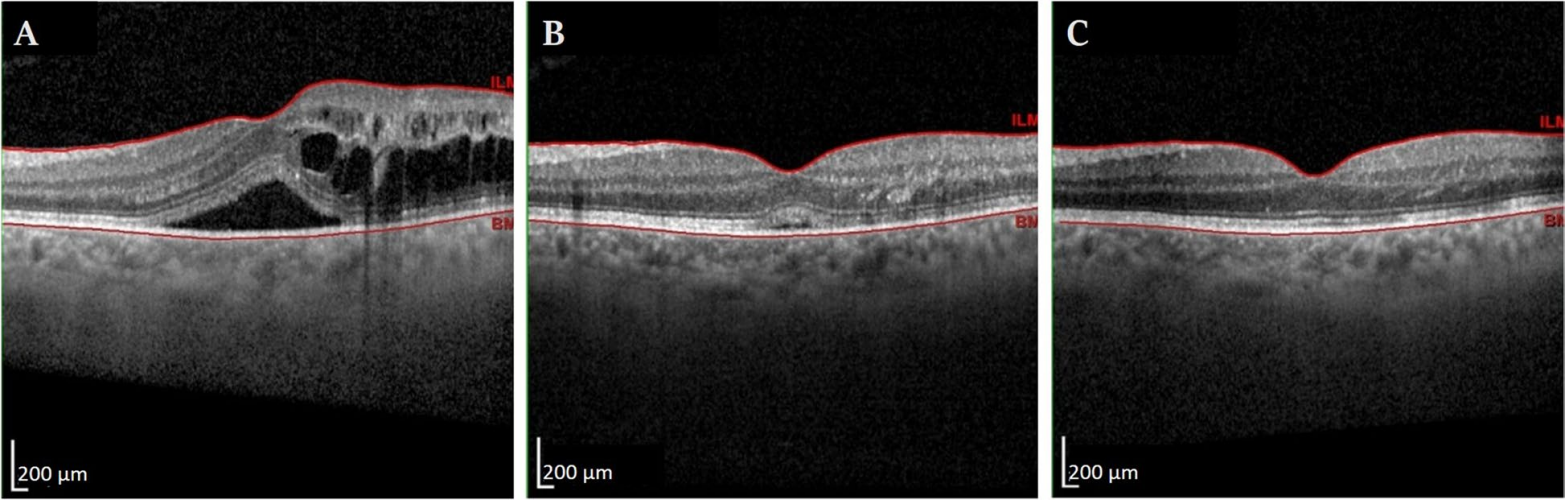
BRVO associated ME in a 63-year-old male presenting with blurred vision for 1 month ago with baseline BCVA of 20/40. **A** Baseline OCT image showing macular edema with intraretinal and subretinal fluid with baseline CST of 506 μm. After 1 week of SCTA injection, BCVA improved to 20/20 and CST decreased to 310 μm with minimal subretinal fluid (**B**), which resorbed completely after 1 month. Complete regression was preserved until the end of follow-up period after 3 months (**C**)

**Fig. 3:**
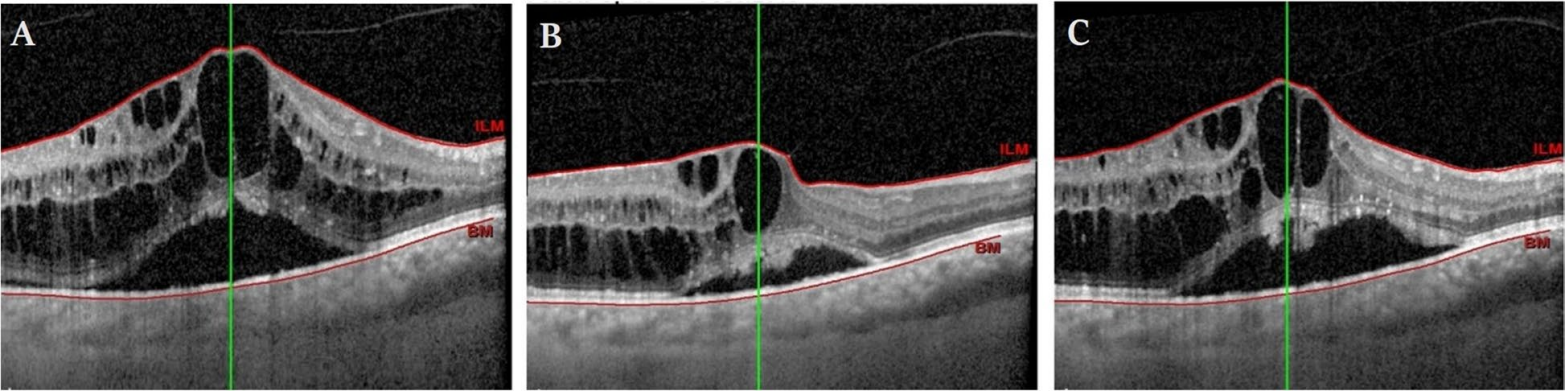
ME associated with CRVO in a 62-year-old female patient presenting with blurred vision for 8 months ago with baseline BCVA of 0.1 and a history of prior treatment with 5 anti-VEGF injections with partial improvement. **A** Baseline OCT image showing severe macular edema with intraretinal and subretinal fluid; baseline CST was 1130 μm. After 1 week of SCTA injection, BCVA improved to 0.32 and CST decreased to 508 μm which increased to 618 after a month (**B**) ME further recurred in the second month after injection with CST of 812 and BCVA of 0.1 (**C**)

The primary efficacy endpoint was the percentage of participants with BCVA gain ≥15 letters in months 1, 2, and 3. Secondary endpoints included mean change from baseline BCVA and CST at months 1, 2, and 3; percentage of participants with CST ≤ 320 mm at months 1, 2, and 3; mean percent reduction in excess foveal thickness (EFT) (EFT was estimated by subtracting 310 μ from the central subfield thickness) at months 1, 2, and 3; and time to the first recurrence of macular edema, an example is shown in Figs. 2 and 3.

Primary safety endpoints included the percentage of participants with IOP ≥20 mmHg through months 1, 2, and 3. Secondary endpoints included the incidence of serious treatment-emergent adverse events including Endophthalmitis, Intraocular inflammation, vitreal hemorrhage, retinal tear, and Rhegmatogenous retinal detachment; and the mean change from baseline IOP in months 1, 2 and 3.

### Statistical analysis

The goal of this pilot study is to assess the feasibility of the suprachoroidal injection of TA using the custom-made injector in terms of safety and potential efficacy and the applicability of a larger-scale Randomized controlled trial (RCT) in light of the lack of previous studies in this regard. A total sample size of 15 was calculated using G Power 3.1 to have 95% power to detect a large effect size (as used and suggested by Campochiaro et al. study [24]) of 0.40 (suggested by Cohen et al. [26]) using F-test ANOVA repeated measures within factors with a 0.05 1-sided significance level. Whitehead et al. [26] have also recommended pilot trials sample sizes per treatment arm of 15 and 10 for medium (0.5) and large (0.8) standardized effect sizes respectively. In view of this, 16 participants were considered as a reasonable sample size to be recruited during the study period and to assess the appropriateness of the applied intervention and the used injector, and to offer estimations about the enrollment rate and the statistical parameters required for future RCTs.

Continuous variables were demonstrated as mean ± SD and categorical variables as frequencies and percentages. Patient Demographics and Baseline Characteristics were compared between BRVO and CRVO patients using 2-sided T-test for continuous variables and Cochran-Mentel-Haenszel (CMH) X2 or Fisher’s exact tests for categorical variables.

Repeated measures ANOVA was applied to compare differences in BCVA letter score, CST, and IOP between baseline and each follow-up visit. Mauchly’s test was used to assess sphericity assumption; in case of violation, the Huynh-Feldt or Greenhouse-Geisser correction factors were considered to get the adjusted *P* values for each univariate F test involving the interval effect. Bonferroni correction was applied to adjust *p*-values for multiple post hoc comparisons. *P* values ≤ .05 were considered significant.

Survival analysis of the first recurrence of macular edema was evaluated using the Kaplan–Meier method, where the recurrence of macular edema (the event being followed) was defined as the presence of macular edema with a central subfoveal thickness of CST > 320 μm with retinal or subretinal fluid, Kaplan Meier was applied for the survival analysis through the 3-month follow up period starting from the first month. Differences between CRVO and BRVO groups were assessed using the log-rank test comparing the cumulative response rate curves across time during the 3-month study period.

SPSS for Windows (Version 26; SPSS Inc., Chicago, IL, USA) was used for statistical analysis. A *p*-value of ≤0.05 was considered statistically significant.

## Results

### Patient demographics, baseline characteristics, and disposition

A total of 16 eyes of 16 patients that met the criteria were included in this study. Baseline characteristics of the enrolled patients are described in Table 1. 10 (62.5%) were BRVO\HRVO patients and 6 (37.5%) were CRVO patients, 7 (56.3%) were males, the mean age of the study group was 55.81 ± 10.035 years, mean duration of onset of symptoms calculated from the first reported reduced visual acuity by the patient was 6.625 ± 8.038 months, 6 patients had a chronic RVO with a mean duration of 14.25 months (range 7.5- 25) with a previous history of treatment with an average of 6,16 intravitreal Bevacizumab injections (range 3 - 14), mean baseline BCVA was 58.13 ± 16.0 letters (0.36 ± 0.17 Snellen equivalent), mean IOP was 15.06 ± 2.816 mmHg, and mean baseline CST was 660.81 ± 221.581. Macular edema was significantly more chronic in CRVO patients with significantly lower baseline BCVA.

**Table 1:** Descriptive statistics for demographics and baseline clinical characteristics of the study participants

Parameter	Total (***N*** = 16)	BRVO/HRVO (***N*** = 10)	CRVO (***N*** = 6)	***P*** value
**Age, yrs, Mean (SD)**	55.81 (10.035)	56.30 (10.5)	55.0 (10.12)	0.812
**Gender**				0.152
**Female, n (%)**	9 (56.3)	7 (70)	2 (33.33)	
**Male, n (%)**	7 (43.8)	3 (30)	4 (66.66)	
**RVO type, n (%)**	16 (100)	BRVO 9 (56.3) HRVO 1 (6.2)	6 (37.5)	
**Hx of DM, n (%)**	3 (18.8)	0 (0)	3 (50)	0.013*
**Hx of HTN, n (%)**	10 (62.5)	7 (70)	3 (50)	0.424
**Hx of previous Tx (Intravitreal Bevacizumab)**	10 (62.5)	5 (50)	5 (83.33)	0.182
**mean duration of RVO-ME symptoms, mos (SD)**	6.625 (8.038)	2.8 (2.37)	13 (10.28)	0.031*
**BCVA, ETDRS letter score, Mean (SD)**	58.13 (16)	56.5 (6.85)	45.83 (19.85)	0.047*
**Snellen equivalent, Mean (SD)**	0.36 (0.17)	0.42 (0.04)	0.25 (0.08)	
**CST, μm, Mean (SD)**	660.8 (221.58)	597.90 (165.56)	765.67 (227.21)	0.148
**IOP, mmHg, Mean (SD)**	15.06 (2,82)	14.9 (3.14)	15.33 (2.42)	0.233
**IOP-lowering medication, n (%)**	3 (18.8)			
**Phakic, n (%)**	16 (100)			

*Abbreviations*: *BRVO* branch retinal vein occlusion, *CRVO* central retinal vein occlusion, *HRVO* Hemi-retinal vein occlusion, *Hx* history, *DM* Diabetes Miletus, *HTN* hypertension, *Tx* treatment, *mos* months, *BCVA* best-corrected visual acuity, *ETDRS* Early Treatment Diabetic Retinopathy Study, *CST* central subfield thickness, *IOP* intraocular pressure, *SD* standard deviation

* *P* < 0.05, statistically significant

### Scleral thickness and the technical aspects of the injection

Mean scleral thickness at the injection site (4 mm away from the limbus in the inferotemporal quadrant) was 824.38 ± 69.375 μm (Range: 720-920) Table 2.

**Table 2:** Descriptive statistics of the baseline scleral thickness measured using AS-OCT (Microns)

Mean	95% CI for mean		Standard deviation (SD)	Minimum	Maximum
	Lower Bound	Upper Bound			
824.38	787.41	861.34	69.375	720	920
**Scleral thickness (microns)**			**[700-800[**	**[800-900[**	**[900-1000[**
Number of patients			5	7	4

*Abbreviations*: *AS-OCT* Anterior Segment Optical Coherence Tomography, *CI* Confidence Interval

In our study we have used the usual available 27-gauge needle with a bevel of ~ 1 mm length, During the injection, a variable length of the bevel remains consequently outside, ranging in our study between ~ 80-280 μm. However, this free part of the bevel remains during injection covered with the sleeve, and therefore the volume that may leak is bounded laterally by the sleeve (with an inner diameter of ~ 0.45 mm and a length of ~ 126 mm) and the needle (with an outer diameter of ~ 0.413 mm) and inferiorly by the ocular surface regarding that the injection occurs perpendicular to the surface of the sclera and might reach superiorly to the base of the needle. This volume is therefore small, as can be shown by calculation, ( ~ 0.0032 mL) and can be neglected.

During the procedure particular attention was taken to avoid applying pressure after penetrating the sclera and it was ensured that the sleeve was relaxed before the injection of Triamcinolone was performed to avoid any compression of the flexible sleeve, and thus the unintentional increase of the needle length.

The injection was carried out under a surgical microscope with mydriasis. Intraocular pressure was checked out and indirect fundoscopy was also performed immediately after the injection to detect the passage of the injected Triamcinolone into the vitreal cavity. We were also able through anterior segment OCT performed immediately after injection to show the presence of triamcinolone in the suprachoroidal space, as shown in Fig. 1.

The injection process was smooth in all patients. No cases of Triamcinolone penetration into the intravitreal cavity were recorded. In all cases, the injection was performed using the pre-prepared syringe without the need to adjust the needle length during injection.

### Main efficacy outcomes

**Fig. 4:**
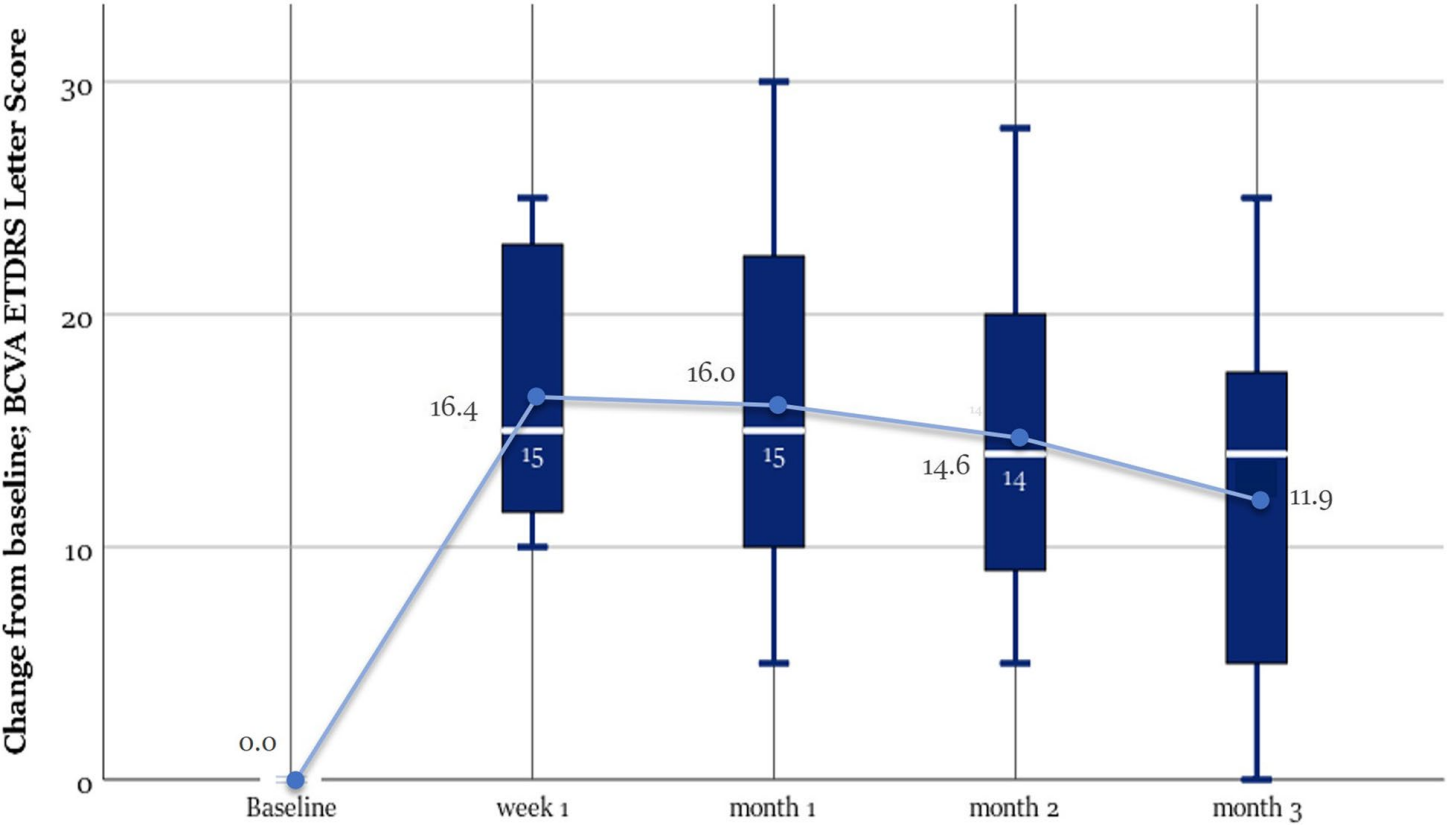
Boxplot graph illustrating the change from baseline Best Corrected Visual Acuity (BCVA) in ETDRS letters score at each visit: The median is marked by the horizontal line inside the box; the circle refers to the mean; Each box spans the interquartile range (IQR) between the 75th, and 25th percentiles. The whiskers extend over a range of 1.5 times the IQR below the 25th percentile and above the 75th percentile, respectively

**Fig. 5:**
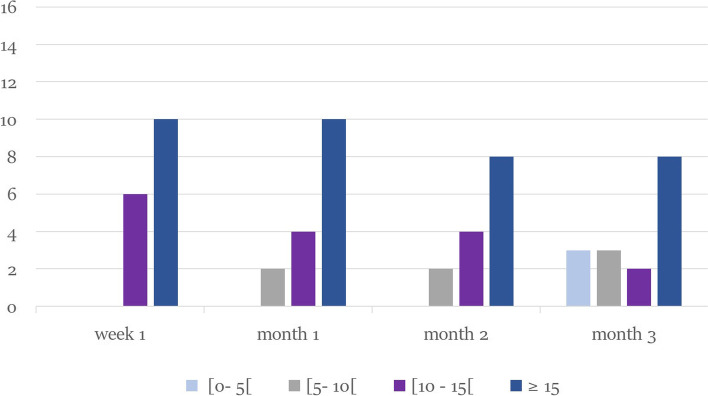
Bar graph showing the number of patients gaining < 5, [5-10[, [10-15[and 15 or more ETDRS letters from baseline through follow-up points

Primary endpoint analysis showed that the percentage of patients with BCVA gain from baseline of 15 or more ETDRS letters was 68.7% (10/16) after 1 week, 62.50% (9/16), 50% (8/16), and 50% (8/16) after 1,2 and 3 months respectively. Mean improvement from baseline letter score were 16.38 ± 5.80 (*p* value< 0.0005), 16.0 ± 7.40, 14.62 ± 7.10, and 11.93 ± 8.25 letters in the first week, first, second and third months respectively, and this improvement was statistically significant in each of the follow-up points compared to the baseline values (*p* value< 0.0005) with no statistically significant difference when compared to each other (*p* values =0.482 - 1.0). The percentages of patients who obtained ≥70 ETDRS letter score (Snellen equivalent ≥0.5) were 81.25, 75, 75, and 62.5% in the first week, first, second and third months respectively Table 2 Figs. 4 and 5.

**Fig. 6:**
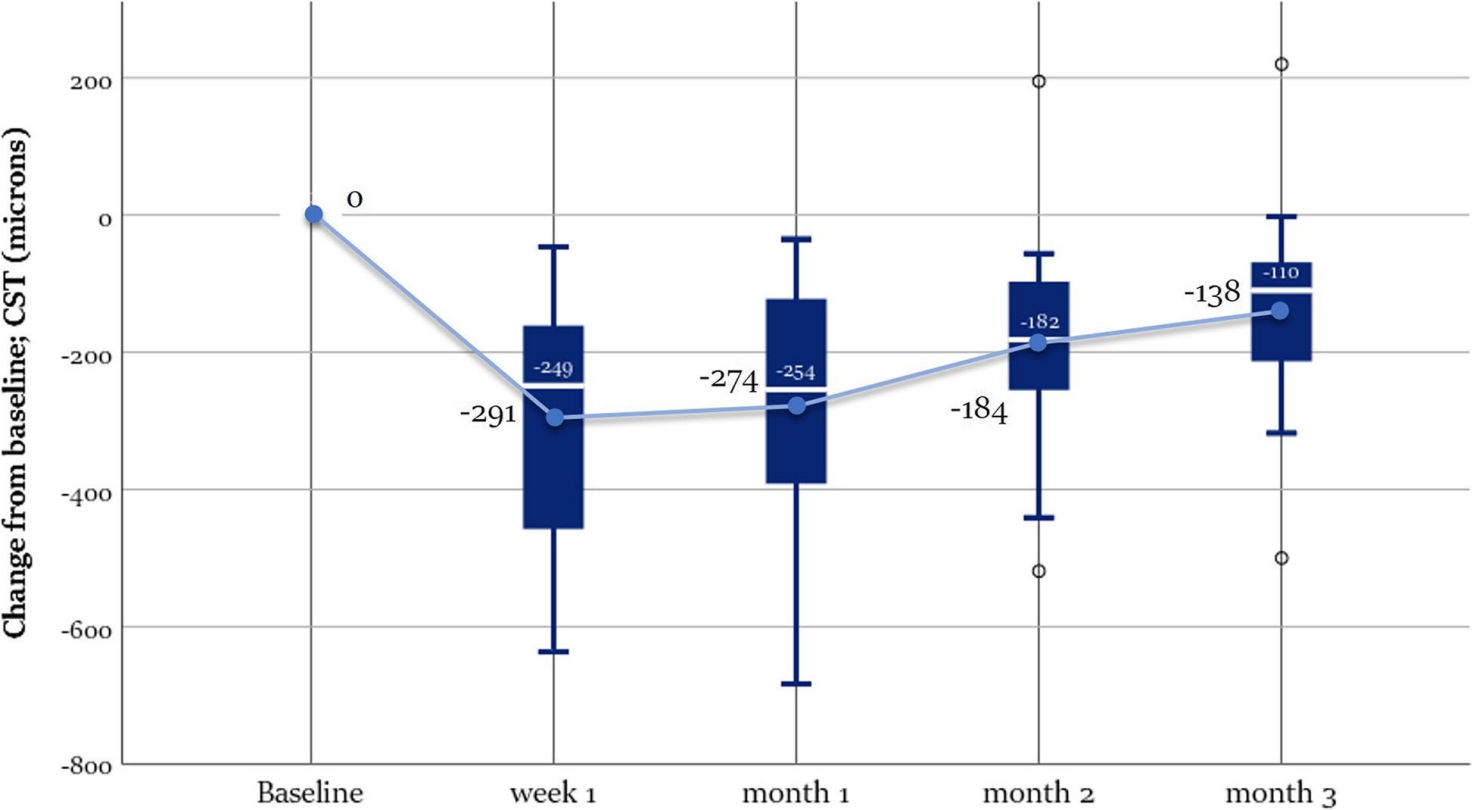
Graph showing the change from baseline in central subfield thickness in microns read at each visit. The median is marked by the horizontal line inside the box; the central circle refers to the mean; Each box spans the interquartile range (IQR) between the 75th, and 25th percentiles. The whiskers extend over a range of 1.5 times the IQR below the 25th percentile and above the 75th percentile, respectively. The outliers (circles) mark any outside the upper and lower fences

This improvement of BCVA was associated with significant reduction of CST by 290.94 ± 181.76 (week 1) (*p* value< 0.0005), 274.31 ± 184.60 (month 1) (*p* value< 0.0005), 183.50 ± 165.61 (month 2) (*p* value = 0.006) and 137,75 ± 156.25 μm (month 3) (*p* value = 0.038). The mean percent reduction in EFT was 89.75 ± 30%, 84.75 ± 35.2%, 57.86 ± 47.54%, 44.67 ± 50.6% through the first week, first, second and third months respectively. This improvement was statistically significant in each of the follow-up points compared to the baseline values, but it decreased significantly in the second month compared to the first one (*p* value = 0.017). The rate of patients achieving CST ≤320 μm was 62.50% at week 1 and 42.75, 18.75, and 18.75% at months 1, 2, and 3 Table 3 Fig. 6.

**Table 3:** Outcome of ophthalmological evaluation at baseline and during 3 months after injection

	BCVA Mean ± SD (letters) Mean ± SD (Snellen equivalent)	*P value	CST Mean ± SD (μm)	*P value	IOP Mean ± SD (mmHg)	*P value
**Baseline**	58.13 ± 16 0.36 ± 0.17	–	660.81 ± 221.58	–	15.06 ± 2.82	–
**Week 1**	74.5 ± 11.58 0.69 ± 0.28	0.000**	369.75 ± 116.26	0.000**	15.81 ± 3.03	0.09
**Month 1**	74.13 ± 13.8 0.70 ± 0.32	0.000**	386.50 ± 110.50	0.000**	15.75 ± 2.89	0.60
**Month 2**	72.75 ± 14.92 0.67 ± 0.33	0.000**	481.81 ± 175.54	0.006**	15.56 ± 2.78	1.00
**Month 3**	70.06 ± 16.5 0.62 ± 0.35	0.001**	526.94 ± 185.61	0.038**	15.56 ± 2.63	0.72
	**Mean BCVA change from baseline (95% CI)**		**Mean CST change from baseline (95% CI)**			
**Week 1**	16.38 (13.28 - 19.47)		−290.94 (− 387.79 - -194.08)			
**Month 1**	16.00 (12.06 -19.94)		−274.31 (− 372.68 - -175.95)			
**Month 2**	14.63 (10.84 - 18.41)		−183.50 (− 271.75 - -95.25)			
**Month 3**	11.94 (7.54 - 16.34)		−137.75 (−221.01 - -54.49)			

*Abbreviations*: *BCVA* best-corrected visual acuity, *CST* central subfield thickness, *IOP* intraocular pressure, *CI* confidence interval

* Repeated measures ANOVA for comparison with baseline

** *P* < 0.05, statistically significant

**Fig. 7:**
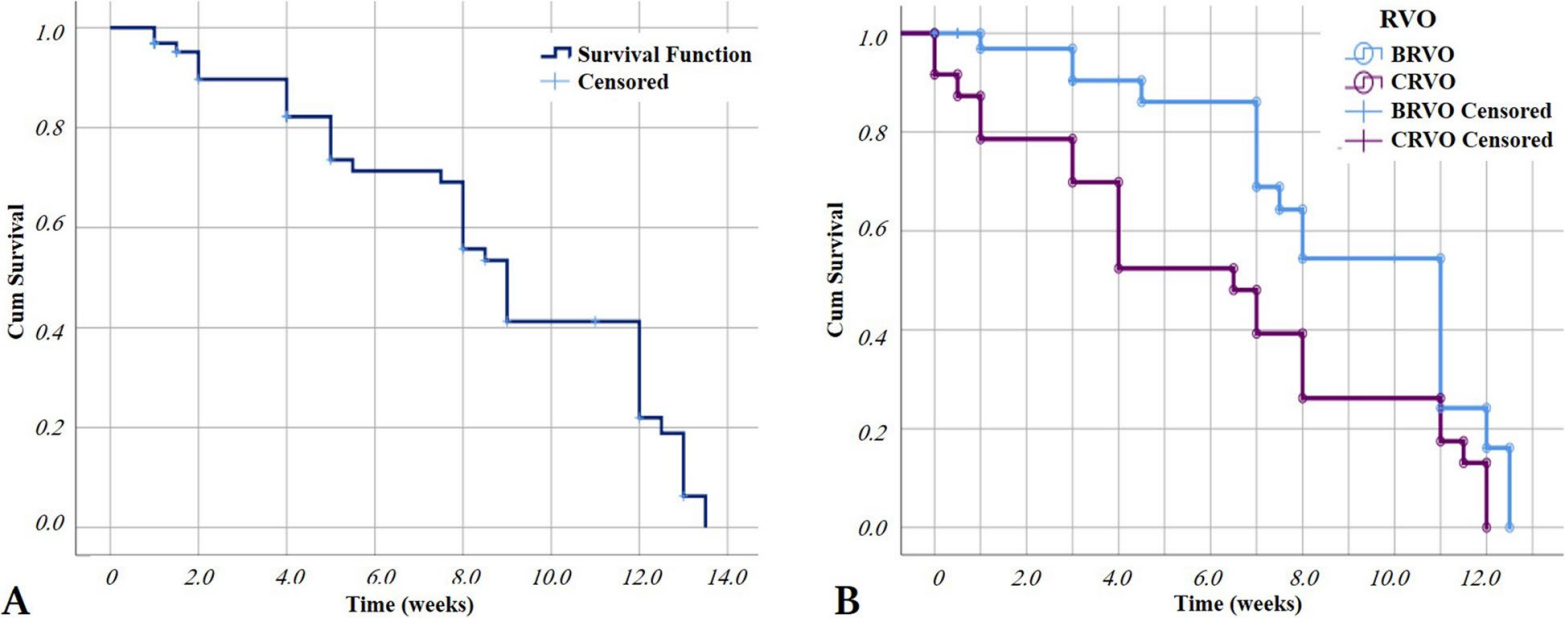
**A**: Kaplan-Meier graph showing time to the first recurrence of macular edema in our group study. **B**: Kaplan-Meier graph showing time to the first recurrence of macular edema in CRVO and BRVO/HRVO groups. The 2 curves represent the cumulative proportion of recurrence-free intervals for each group

The time to the first recurrence was 8.73 ± 0.55 weeks (mean ± SD) in the whole group, 10.07 ± 0.64 weeks (mean ± SD) in the BRVO group, compared with 7.05 ± 0.87 weeks in the CRVO group. The CRVO group had a statistically significant shorter interval to the first recurrence. (Log-rank test, *p* = 0.012) Fig. 7.

### Main safety outcomes

**Fig. 8:**
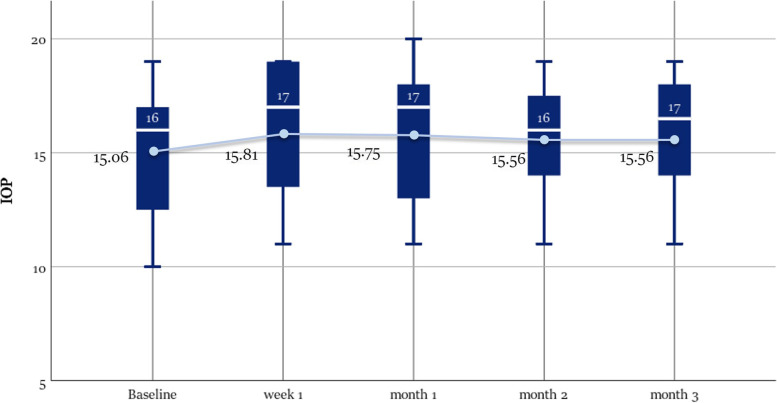
Boxplot Graph showing the change from baseline in IOP in mmHg at each visit. The median is marked by the horizontal line inside the box; the circle refers to the mean; Each box spans the interquartile range (IQR) between the 75th, and 25th percentiles. The whiskers extend over a range of 1.5 times the IQR below the 25th percentile and above the 75th percentile, respectively

We have not reported any of the serious adverse events (SAE) during follow-up period. The mean change of IOP readings was not statistically significant, with an increase ranging from 0.75 mmHg after the first week (*p*-value = 0.09) and 0.5 mmHg after 3 months (*p*-value = 0.72). There was 1 participant (6.25%) who had increased intraocular pressure from 17 mmHg before injection to 20 mmHg after 1 month that was normalized in the second month Fig. 8.

## Discussion

Blockage of the venous drainage through the central retinal vein or any of its major branches compromises perfusion to the whole or a sector of the retinal tissue, resulting in retinal ischemia and upregulation of hypoxia-stimulated genes, particularly important is the one that encodes vascular endothelial growth factor (VEGF) [3]. Acting as a pro-angiogenetic and pro-permeability factor, VEGF represents a major promotor of ME associated with RVO, but not the only key player. It was recently further emphasized to be highly impacted by other inflammatory mediators as a result of hypoxia-parallel multiple inflammatory cascades [4–6], suggesting an additive important role of inflammation that deserves to be taken into therapeutic consideration [6].

Management of macular edema was revolutionized by the development of different anti-VEGF agents, which represent now the first-line therapy [7]. The efficacy of anti-VEGF agents was well documented in phase III, multicenter, randomized studies [8–12]. with a mean BCVA gain of + 18.3 and + 14.9 letters and mean change in CRT from baseline of − 354 and 452 μm in BRAVO and CRUISE studies after 6 months of treatment with intravitreal Ranibizumab 0.5 mg in BRVO and CRVO patients respectively [8, 9]..

MARVEL study has shown comparable results of monthly injected 1.25 mg Bevacizumab with mean BCVA gain of + 15.6 letters and mean change in CRT from baseline of − 201.7 μm after 6 months of treatment [10].

The efficacy of Aflibercept has also been proven in the COPERNICUS /GALILEO and VIBRANT Studies with a mean BCVA gain of + 17.7 and + 17 letters and a mean reduction of CRT of − 453 and–280.5 μm after 6 months of monthly injected 2 mg Aflibercept in CRVO and BRVO patients respectively [11, 12].

Although the early outcomes are remarkable in most patients, they often require persistent treatment and some of them experience suboptimal response [16, 17]. As reported in the RETAIN study, 50 and 56% of BRVO and CRVO patients respectively were still in need of further injections to control macular edema after 4 years of follow-up [16];. Spooner et al. have, moreover, reported that the number of anti-VEGF injections did not seem to show a decreasing trend between the second and fifth years of follow-up, in contrast to eyes with diabetic macular edema [17]. A recent ‘Real-World’ analysis of 15,613 patient eyes also highlighted that patients receive less anti-VEGF injections and experience approximately 10 letters less visual gain in contrast to patients undergoing anti-VEGF therapy based on randomized controlled trials (RCTs) protocols [18], all of which suggest unmet needs associated with treatment burden.

Corticosteroids have theoretically a more favorable therapeutic profile due to their anti-inflammatory, anti-permeability and angio-static properties, as they cause downregulation and repression of gene transcription of many pro-inflammatory mediators, adhesion molecules as well as VEGF, targeting a wider spectrum of factors involved in the pathophysiology of RVO associated ME with prolonged duration of action [7, 27].

TA was the first steroid to be injected intravitreally as a readily available pharmacologic agent, though its use for the treatment of macular edema is off-label. The most extensive clinical trial investigating TA as a treatment of RVO associated ME was the SCORE (Standard Care vs. Corticosteroid for Retinal Vein Occlusion) multicenter clinical trial. After 12 months of 4-monthly injected TA, it has been shown that 27 and 26% of participants achieved a visual gain ≥15 letters in the 4-mg group in BRVO and CRVO patients, respectively. However, IVTA was associated in this study with the need for IOP-lowering medications in 35 and 41% of patients as well as development or progression of lens opacity in 33 and 35% of phakic patients in the 4-mg group in BRVO and CRVO studies, respectively [7, 13, 14].

In 2009, a sustained-release intravitreal 0.7 mg dexamethasone (Ozurdex®) delivery system was approved by the FDA to treat ME secondary to RVO [7]. Its efficacy was proved through GENEVA study, which reported a peak visual gain of + 10 letters after 2 months. After 6 months, the mean BCVA gain was + 5.2 letters; the rate of 15-letter BCVA gain from baseline was 25% of patients and the mean reduction of CRT was − 123 μm in 0.7 mg groups. There was an associated increased IOP of ≥25 mmHg which peaked at 16% at day 60. Cataract has also developed in 7.2% of phakic patients after 6 months [15].

A recent meta-analysis of 6 randomized clinical trials (RCT) in RVO patients showed that IVT injection of TA, no more frequently than 4-monthly, has comparable visual and more significant outcomes to anti-VEGF agents [28]. Another meta-analysis of 7 RCTs “found no evidence of differences between ranibizumab, aflibercept, bevacizumab, and triamcinolone for improving vision in CRVO patients” [29]. However, the high incidence of adverse events, including ocular hypertension and/or cataract in phakic patients has limited its consideration as an option early in the course of RVO [28, 29].

Suprachoroidal drug delivery provides an advantageous novel method to deliver steroids by dominantly targeting affected tissues that may boost efficacy, compartmentalizing therapeutic agent away from unaffected tissues, thereby minimizing the risk of cataract and increased IOP which enhances safety, and also achieving durability to reduce the treatment burden noted with other current agents [20–22]. Our study further supports this hypothesis, as it showed that this treatment provided a significant visual gain of 16.4, 16, 14,6, and 11.9 letters in week 1 and months 1, 2, and 3 respectively with 50% of patients gaining 15 or more ETDRS letters through month 3. These visual results were accompanied by a significant reduction of CST, though the latter appeared to be less persistent. EFT was reduced by a mean of 89.75 and 84.75% and CST was normalized in 62.50 and 42.25% of patients in the first week and first month, respectively. However, there was a significant recurrence in the second month with a mean EFT reduction of only 57.86 and 44.67% of patients and complete ME regression in 18.75% of patients at months 2 and 3, respectively.

Survival analysis in our study showed that the time to the first recurrence was 8.7 weeks. Treatment efficacy appeared to be significantly more prolonged in BRVO patients by a difference of about 3 weeks (10 vs. 7 weeks to the first recurrence in BRVO vs. CRVO patients). However, it is worth noticing that ME was more chronic in CRVO patients in our study group (Mean duration of RVO-ME symptoms 2.8 ± 2.37 months in BRVO vs 13.0 ± 10.28 months in CRVO patients) with worse baseline BCVA ETDRS letter score (56.5 ± 6.85 letters in BRVO vs 45.83 ± 19.85 letters in CRVO patients).

Regarding safety, our study showed that mean IOP did not change significantly over a 3-month follow-up period. One participant with no previous history of glaucoma experienced increased IOP to 20 mmHg that has reduced to 18 in the second month. We did not report the occurrence of any SAE as well. Development or progression of cataract was also not noticed at the end of the 3 month follow up period. However, this measure was not addressed in the safety measures in our study due to the short follow-up duration.

To the best of our knowledge, none of the previous studies evaluated the efficacy and safety of suprachoroidal TA (SCTA) as a mono-treatment of RVO associated ME. A phase 2 study (TANZANITE) evaluated the safety and effectiveness of suprachoroidal injection of suprachoroidal triamcinolone acetonide (CLS-TA), an exclusive investigational suspension of triamcinolone acetonide, in combination with IVT aflibercept in patients with ME secondary to RVO [24]. A total of 46 subjects with RVO were randomly assigned by a ratio of 1:1 to either suprachoroidal injection of CLS-TA plus IVT aflibercept or IVT injection of aflibercept alone. Participants in each study group received PRN aflibercept at Months 1, 2, and 3 on as needed basis. The key hypothesis evaluated was whether the additional suprachoroidal CLS-TA caused prolonged resolution of macular edema, thereby decreasing the need for additional IVT aflibercept injection during this 3-month follow-up period. This phase 2 trial reached its primary endpoint. Compared with aflibercept monotherapy, the number of aflibercept treatments required for the combination therapy was significantly reduced (23 versus 9; 61% reduction; *p* = 0.013) with lower rate of participants who needed re-treatment (78% versus 30%; *p* = 0.003). Mean visual acuity gains were 16, 20, and 19 letters in the combination arm and 11, 12, and 11 letters in the aflibercept arm, in the first, second, and third months respectively; the difference between the two groups was statistically significant in the second month (*p* = 0.04). Anatomic improvement was more remarkable in the combination arm in Month 3 (285.4 μm versus 384.6 μm). Increased IOP measurements was reported in 4 subjects in the combination group versus no patient in the monotherapy arm. Otherwise, ocular adverse events were similar between the two treatment arms [24]. Extension data for additional 6 months of follow-up were collected and analyzed retrospectively, and the results showed that the combination therapy reduced the need of additional aflibercept treatment by about 68% (6 out of the 23 subjects (26%) in the combination therapy compared to 19 out of 23 subjects (83%) in aflibercept monotherapy arm required additional treatment with IVT aflibercept during the 9-month follow-up period) [22, 30].

The phase 3 SAPPHIRE clinical trial investigated the superiority of CLS-TA\Aflibercept combination treatment, compared to the monotherapy with IVT Aflibercept. This study failed to achieve its primary endpoint, as the proportion of subjects with improvement from baseline of ≥15 ETDRS letters in week 8 was approximately the same (~ 50%) in each treatment group; consequently, the study sponsor suspended the development of suprachoroidal CLS-TA in combination with aflibercept for the indication of ME due to RVO [22, 31].

Delivery to the SCS via microneedles exhibit convenient pharmacodynamics preclinically as it provides posterior and circumferential distribution of agents injected anteriorly [20, 23] and it has been shown to be safe and effective clinically in Phase 3 trials of CLS-TA using microneedle-based technology, the SCS Microinjector® (Clearside Biomedical, Alpharetta, GA), which is supplied with two 30G microneedles with fixed free lengths of 900 or 1100 μm [22, 24, 25].

Reviewing over 1000 injection procedures performed over 6 clinical trials, Wan et al. demonstrated that 84% of physicians did not perceive the﻿ SC injections to be more challenging than other usual ocular injections. However, 29% of injections could not be performed using a 900 μm needle, and switching to the longer needle was required. This percentage was correlated with the injection quadrant (22% of superotemporal quadrant injections versus 35% of inferotemporal quadrant injections) [25].

Scleral thickness (ST) at the injection point in pars plana is a key anatomical player for the success of such type of ocular injection as the sclera must be precisely penetrated to avoid piercing through choroidal tissues [20]. The thickness of the sclera and surrounding tissues can be non-invasively and reliably measured using AS-OCT, which is increasingly integrated into clinical practice. AS-OCT based studies showed that average ST at 4 mm away from the limbus measures 716 ± 52 μm [32], and indicated that significant variations in ST occur with regard to meridian, distance from the limbus, age, and gender [32, 33].

In our study, we depended on the measured thickness of scleral and surrounding tissues in the inferotemporal quadrant with a mean thickness of 824.38 ± 69.375 μm (Range: 720-920) using AS-OCT to design a patient-customized low-cost microinjector. Injection procedures were easily performed without encountering any significant unique difficulties. There were no documented instances of Triamcinolone penetrating the intravitreal cavity, and all injections were carried out using the pre-prepared syringe without the need to adjust the needle length during injection.

The cost per dose of suprachoroidal injection of preserved TA in our study was low compared with that of IVT Bevacizumab, Ranibizumab, or Aflibercept, which are about 7, 250, and 225 times higher, respectively. Thus, SCTA could be highly beneficial in low-income countries with deficient insurance coverage, where the treatment options are markedly limited, due to the high costs and shortage of supply.

The main limitations of this study are the small study group, the short duration of follow-up (3 months), and the non-comparative study design. Visual outcomes in our study appeared to be more persistent compared with anatomical outcomes, as the latter showed a more heterogeneous response pattern, especially in the second and third months. This suggests that factors in addition to changes in central retinal thickness could be affecting visual acuity in RVO associated ME and reflects the importance of studying the potential effect of baseline characteristics and OCT biomarkers in futuristic studies, that could be beneficial in identifying more responsive patterns and others that need a more frequent or combined therapy regimen.

## Conclusion

This preliminary study suggests that suprachoroidal injection of TA could be an efficacious, tolerable, and low-cost therapeutic alternative for the treatment of RVO associated macular edema. However, Future randomized clinical trials with larger sample size and more extended follow-up period are needed to confirm these results over the longer term and to compare its efficacy and safety profile with other treatment options.

## Data Availability

The datasets generated during and/or analyzed during the current study are not available publicly due to privacy and ethical concerns but are available from the corresponding author on reasonable request.

## Abbreviations

AS-OCT: Anterior Segment Optical Coherence Tomography
BCVA: Best-Corrected Visual Acuity
BRVO: Branch Retinal vein occlusion
CMH: Cochran-Mentel-Haenszel
CRVO: Central Retinal Vein Occlusion
CST: Central Subfield Thickness
EFT: Excess Foveal Thickness
ETDRS: Early Treatment Diabetic Retinopathy Study
HRVO: Hemi-Retinal Vein Occlusion
IRB: Institutional Review Board
ME: Macular Edema
RCTs: Randomized Controlled Trials
RVO: Retinal vein occlusion
SAE: Serious Adverse Events
SCORE: Standard Care vs. Corticosteroid for Retinal Vein Occlusion
SCS: Suprachoroidal Space
SCTA: Suprachoroidal triamcinolone acetonide
SD: Standard Deviation
SD-OCT: Spectral Domain Optical Coherence Tomography
ST: Scleral thickness
TA: Triamcinolone Acetonide
VEGF: Vascular Endothelial Growth Factor
